# Correlation Between Serum Vitamin D Levels and the Severity of Coronary Artery Disease in Patients With Myocardial Infarction

**DOI:** 10.7759/cureus.93138

**Published:** 2025-09-24

**Authors:** Bisma Naveed, Syeda Ammara Fatima, Fatima Choudhry, Prathik Saravanan, Aarib Ahmed, Nabiha Sahar, Taimoor Shahid, Hammad Yousaf, Muhammad Ibaad Siddiqui, Zoya Usman, Areeba Shoaib

**Affiliations:** 1 Cardiology, Jinnah Hospital, Lahore, PAK; 2 Internal Medicine, Punjab Rangers Teaching Hospital, Lahore, PAK; 3 Internal Medicine, Karachi Institute of Medical Sciences, Karachi, PAK; 4 Internal Medicine, Government Thiruvannamalai Medical College and Hospital, Thiruvannamalai, IND; 5 Stroke Medicine, University Hospitals Coventry and Warwickshire NHS Trust, Coventry, GBR; 6 Internal Medicine, Jinnah Hospital, Lahore, PAK; 7 Internal Medicine, Shalamar Medical and Dental College, Lahore, PAK; 8 Cardiology, University Hospitals Coventry and Warwickshire NHS Trust, Coventry, GBR; 9 Internal Medicine, Rashid Latif Medical College, Lahore, PAK

**Keywords:** artery, coronary, correlation, disease, infarction, myocardial, serum, severity, vitamin d

## Abstract

Background: Coronary artery disease (CAD) is one of the major contributors to death around the globe. This study aimed to investigate the relationship between serum vitamin D levels and the severity of CAD in patients with myocardial infarction (MI).

Methods: This cross-sectional analysis was carried out on 285 patients with MI at the Cardiology Unit of Jinnah Hospital, Lahore, Pakistan, from September 2022 to September 2023. All patients were enrolled through consecutive sampling and predetermined inclusion and exclusion criteria. A self-designed proforma was used for data collection. The patients were divided into two categories according to their CAD severity, using the Gensini score system: non-severe CAD and severe CAD. A comparative analysis between patients with non-severe CAD and severe CAD was performed via independent t-tests and chi-square tests. Pearson's correlation coefficient measured the relationship between serum vitamin D levels and CAD severity. Linear regression analysis evaluated the predictive value of serum vitamin D level for CAD severity. A p-value below 0.05 was set as statistically significant. This data analysis was conducted using IBM SPSS Statistics for Windows, Version 25 (Released 2017; IBM Corp., Armonk, New York, United States).

Results: Among the 285 patients, 65.95% (n=188) had non-severe CAD, while 34.05% (n=97) had severe CAD. Patients with severe CAD showed significantly lower mean serum vitamin D levels (14.60 ± 4.91) compared to those with non-severe CAD (28.85 ± 7.98). Statistically significant variations were noted in Gensini scores (p=0.001) and serum vitamin D levels (p=0.002) between the two study cohorts. A strong negative correlation was found between serum vitamin D levels and CAD severity (-r=0.79, 95% CI=-0.75 to -0.85, p<0.001). Moreover, regression analysis validated the use of serum vitamin D level as a significant determinant of CAD severity, with negative values of unstandardized coefficient (-3.50), standardized coefficient (-0.79), and a 95% CI of -1.92 to -5.40 (p<0.001), and an R-squared value of 0.86.

Conclusions: This study observed a significant inverse relationship between vitamin D levels and CAD severity in patients with MI. Lower vitamin D levels were associated with more severe CAD, suggesting its potential as a risk factor and clinical tool for identifying high-risk patients and facilitating timely interventions, including vitamin D supplementation and other cardiovascular risk-reducing strategies.

## Introduction

Coronary artery disease (CAD) is a major global health concern, ranking among the top causes of illness and death worldwide. CAD develops when the coronary arteries become narrow or blocked due to atherosclerosis. Narrowing or blockage of the arteries leads to decreased blood flow to the heart muscle. Its symptoms could be sudden pain in the chest, shoulder, and jaw, along with dyspnea and sweating [1]. CAD can present in various forms, including acute coronary syndrome (ACS) and chronic coronary syndrome. ACS encompasses three main subtypes: unstable angina, ST-elevation myocardial infarction (STEMI), and non-ST-elevation myocardial infarction (NSTEMI). NSTEMI and STEMI represent acute myocardial infarction (MI), which is a severe and potentially life-threatening form of ACS [2].

The burden of CAD is rapidly growing in both affluent and resource-constrained nations, with younger populations in low- and middle-income countries like Pakistan being disproportionately affected [3]. According to the World Health Organization (WHO), cardiovascular diseases are responsible for a staggering 43% of non-communicable disease-related deaths all over the world. Globally, CAD claimed 17.9 million lives in 2019, predominantly in low- and middle-income countries, and is expected to reach 23.6 million deaths by 2030 [4,5]. Pakistan is particularly hard hit, with an estimated 18.90% of its population suffering from CAD. Furthermore, a Global Burden of Disease study revealed that the age-standardized incidence of cardiovascular disease in Pakistan exceeded the global average in 2019, underscoring the urgent need for effective prevention and management strategies [4,6].

In addition to well-established risk factors for CAD, such as abnormal lipid profiles, high blood pressure, diabetes mellitus, family history of heart disease, smoking, unhealthy diet, and sedentary lifestyle, emerging evidence from both animal and human studies suggests that vitamin D deficiency may play a significant role in the development of CAD and increase CAD severity [7-9]. Given the severity of the CAD epidemic, it is crucial to conduct comprehensive research to identify new risk factors that could serve as potential therapeutic targets for the treatment and prevention of this disease [10].

The pathophysiology of CAD is based on atherosclerosis, a chronic inflammatory process that builds up plaque in the coronary arteries, narrowing them and restricting blood flow to the heart. This process involves immune cells, inflammatory signals, and lipid interactions that lead to damage to the arteries [2,11]. Vitamin D deficiency promotes atherosclerosis by increasing cholesterol uptake by macrophages and foam cell formation, and subsequently causes an increase in the risk of CAD and its severity. Lower vitamin D levels also reduce good high-density lipoprotein (HDL) cholesterol, which further increases the plaque buildup and cardiovascular risk. Moreover, vitamin D deficiency also elevates blood pressure via effects on the renin-angiotensin-aldosterone system and atrial stiffness. Lower vitamin D levels also cause a rise in insulin resistance that causes high blood glucose levels. Raised blood pressure and blood glucose levels further exacerbate the risk and severity of CAD [12-14]

Research in Pakistan reveals a high prevalence of vitamin D deficiency, affecting up to 73% of the population. Major causes of high prevalence of vitamin D deficiency in Pakistan included insufficient exposure to sunlight, poor dietary intake due to poverty, and lack of awareness about its sources and benefits [15]. Although several studies have shown an inverse relationship between vitamin D levels and CAD, suggesting that low vitamin D status may increase the risk and severity of CAD [16,17]. However, a randomized clinical trial on vitamin D supplementation has yielded inconsistent results as well [18]. In the presence of conflicting findings about the correlation between CAD and serum vitamin D levels, further research is needed to obtain the exact role of vitamin D in the development of CAD and its progression, especially in resource-limited countries where research on this correlation is limited.

In Pakistan, research on the correlation between serum vitamin D levels and the severity of CAD is lacking. Therefore, this study aims to investigate the relationship between vitamin D levels and CAD severity in patients with acute MI. By exploring this association, this research seeks to provide valuable insights that will guide clinical decision-making, healthcare policy, and future studies, ultimately improving the prevention and treatment of CAD in this vulnerable population.

## Materials and methods

Study design and study population

This cross-sectional study was performed at the Cardiology Unit of Jinnah Hospital (JH), Lahore, Pakistan, spanning a year from September 2022 to September 2023. We enrolled 285 patients with acute MI who had a complete clinical record of coronary angiography, selected via consecutive sampling based on specific eligibility criteria. The sample size was calculated using the OpenEpi calculator (Open Source Epidemiologic Statistics for Public Health, The OpenEpi Project, Atlanta, GA, United States), based on a prior study that reported a 17.50% prevalence of CAD, with a 95% confidence interval and a 5% margin of error [19]. Prior to the study, we obtained institutional ethics approval (approval JH.ERB.28618) and informed consent from all participants.

Inclusion and exclusion criteria

This study enrolled patients of both sexes, aged 18 and above, with a confirmed diagnosis of ACS based on ECG, cardiac biomarkers, and coronary angiography, and who had a comprehensive medical history. Patients with a past history of cardiac diseases (congenital heart defects, heart failure, and ischemic heart disease) or cardiac intervention, chronic renal or hepatic disease, low hemoglobin, immune system disorders, ongoing infections, cancers, and recent procedures (within four months) were excluded. These exclusions helped minimize confounding variables, ensuring that observed changes were attributed to ACS rather than underlying conditions.

Primary and secondary objectives

The primary aim of the study was to investigate the correlation between serum vitamin D levels and CAD severity, as measured by the Gensini score [20]. Secondary objectives included comparing vitamin D levels in patients with severe CAD versus non-severe CAD and evaluating vitamin D's potential as a predictor of CAD severity.

Evaluation of study variables

MI was diagnosed based on American Heart Association criteria, which included prolonged chest pain (at least 30 minutes duration), characteristic ECG changes, and elevated cardiac enzymes [5]. CAD was diagnosed via coronary angiography, with significant stenosis (>50% vessel diameter) considered diagnostic. CAD severity was assessed using the Gensini scoring system, which has been validated and used in several international studies. It calculates scores based on the stenosis severity and lesion location in the coronary vasculature. Patients were categorized as having non-severe CAD (Gensini score ≤50) or severe CAD (Gensini score >50) [5,13]. To ensure accuracy, two cardiologists independently validated the Gensini scores using standardized guidelines. At admission, levels of serum lipids and vitamin D (using the radioimmunoassay (RIA) method) were measured from blood samples as per hospital protocols. Serum vitamin D levels above 30 ng/mL were considered to be normal, while levels less than 20 ng/mL were viewed as vitamin D deficiency [7].

Data collection

Data was gathered using a self-structured proforma, which had two sections (Appendix 1). The first section captured demographic and clinical information, including age, gender, and the presence or absence of conventional risk factors for CAD, such as family history of CAD, hypertension, diabetes mellitus, dyslipidemia, and smoking history. The second section documented the results of diagnostic tests, including serum vitamin D levels, lipid profiles, cardiac biomarkers, ECG findings, and coronary angiography results, all of which were conducted at JH as part of patient management.

Data analysis

Data analysis was conducted using IBM SPSS Statistics for Windows, Version 25 (Released 2017; IBM Corp., Armonk, New York, United States). Descriptive statistics, including frequencies and percentages, were used to summarize categorical variables, whereas continuous variables were expressed as mean ± standard deviation. Parametric tests were applied due to the normal distribution of the data, which was confirmed by the Shapiro-Wilk test. The quantitative and categorical variables were compared between the two groups via an independent t-test and a chi-square test, respectively. The association between Gensini scores and vitamin D levels was assessed using Pearson's correlation coefficient. Moreover, a linear regression analysis was performed to assess the predictive value of vitamin D levels on Gensini scores. A p-value below 0.05 was considered statistically significant.

## Results

Among 285 patients, n=188 (65.95%) were diagnosed with non-severe CAD, whereas n=97 (34.05%) had severe CAD. The average values (± standard deviation) for key factors were age (58.61 ± 18.89 years), Gensini score (48.93 ± 35.69), and serum vitamin D levels (17.98 ± 7.90 ng/mL).

Table 1 summarizes the baseline and clinical features of the study group. It also reveals significant variations between the non-severe CAD and severe CAD groups in main variables such as Gensini score and serum vitamin D levels (p<0.05). Additionally, although severe CAD was more prevalent among patients with certain contributing factors, including older age, male gender, family history of CAD, high blood pressure, diabetes mellitus, high cholesterol, and smoking history, no statistically significant variations were observed in the pattern of these predisposing factors between the two study cohorts (p>0.05).

**Table 1:** Baseline and clinical characteristics of the study group. N: study population size; n: sample size for each group or category; %: percentage; SD: standard deviation [5,13]: Reference studies in which the Gensini scoring system has been used for the assessment of the severity of coronary artery disease. The table shows test statistics and p-values for two tests, marked as follows: * for independent t-tests and + for chi-square tests, applying to both test statistic values and p-values.

Variables N=285		Display of Variables	Study Groups Based on Coronary Artery Disease Severity		Independent t-test/ Chi-Square test	
					Test Statistics	
			Non-severe Coronary Artery Disease Group n=188 (65.95%)	Severe Coronary Artery Disease Group n=97 (34.05%)	t-value for Independent-Test/ χ²-value for Chi-Square Test	p-values
Age (Years) (Means ± SD)		58.61 ± 18.89	57.50 ± 14.60	59.24 ± 17.68	0.84^*^	0.16^*^
Gensini score (Means ± SD) [5,13]		48.93 ± 35.69	26.76 ± 14.65	72.70 ± 35.45	12.23^*^	0.001^*^
Serum vitamin D level (ng/mL) (Means ± SD)		17.98 ± 7.90	28.85 ± 7.98	14.60 ± 4.91	18.58^*^	0.002^*^
Gender	Male n (%)	170 (59.65)	110 (58.51)	60 (61.86)	0.30^+^	0.90^+^
	Female n (%)	115 (40.35)	78 (41.49)	37 (38.14)		
Family history of CAD	Yes n (%)	90 (31.58)	38 (20.22)	52 (53.60)	3.45^+^	0.06^+^
	No n (%)	195 (68.42)	150 (79.78)	45 (46.40)		
Hypertension	Yes n (%)	198 (69.47)	128 (68.10)	70 (72.16)	0.53^+^	0.60^+^
	No n (%)	87 (30.53)	60 (31.90)	27 (27.84)		
Diabetes mellitus	Yes n (%)	158 (55.43)	104 (55.31)	54 (55.68)	0.14^+^	0.80^+^
	No n (%)	127 (44.57)	84 (44.69)	43 (44.32)		
Dyslipidemia	Yes n (%)	145 (50.88)	96 (51.08)	49 (50.52)	0.12^+^	0.98^+^
	No n (%)	140 (49.12)	92 (48.92)	48 (49.48)		
Smoking history status	Yes n (%)	122 (42.80)	54 (28.72)	68 (70.10)	4.52^+^	0.05^+^
	No n (%)	163 (57.20)	134 (71.28)	29 (29.90)		

Table 2 presents a strong negative correlation between serum vitamin D levels and the severity of CAD, as determined by Pearson's correlation analysis. This association suggests that higher Gensini scores are associated with lower serum vitamin D levels, indicating an inverse relationship between CAD severity and vitamin D levels.

**Table 2:** Correlation between serum vitamin D levels and the coronary artery disease severity. [5,13]: Reference studies in which the Gensini scoring system has been used for the evaluation of the severity of coronary artery disease.

Variables of Patients With Myocardial Infarction N=285	Study Groups Based on Coronary Artery Disease Severity		Independent t-test		Pearson’s Correlation		
			Test Statistics		Test Statistics		
	Non-severe Coronary Artery Disease Group	Severe Coronary Artery Disease Group	t-value	p-value	Correlation Coefficient (r)	95% CI	p-value
Gensini score [5,13]	26.76 ± 14.65	72.70 ± 35.45	12.23	0.001	-0.79	-0.75 to -0.85	0.001
Serum vitamin D level (ng/mL) (Means ± SD)	28.85 ± 7.98	14.60 ± 4.91	18.58	0.002			

Table 3 shows that the linear regression analysis revealed a strong predictive model, with a coefficient of determination (R²) of 0.86 and a highly significant p-value (p<0.001). This indicates a substantial inverse relationship between serum vitamin D levels and Gensini scores. Specifically, the negative regression coefficient suggests that lower vitamin D levels are closely linked to higher Gensini scores, which in turn indicate more severe CAD.

**Table 3:** Analysis of the association between vitamin D levels and CAD severity using a linear regression model. CI: confidence interval; CAD: coronary artery disease

Variable		Test Statistics for the Simple Linear Regression Model					
	Unstandardized Coefficient		Standardized Coefficient	95% CI	p-value	R^2 ^value	p-value of F test
Serum vitamin D level	-3.50		-0.79	-1.92 to -5.40	0.001	0.86 (86.00%)	0.000

## Discussion

CAD is a significant cause of death globally. Early diagnosis, risk stratification, and intervention can substantially reduce CAD-related mortality. While traditional risk factors like hypertension, diabetes mellitus, obesity, sedentary lifestyle, family history, and smoking are well-established, emerging evidence suggests that serum vitamin D deficiency is a significant risk factor for CAD. Vitamin D deficiency may contribute to CAD incidence and severity by promoting atherosclerosis directly or indirectly through increased blood pressure and glucose levels [8-10]. By understanding the relationship between serum vitamin D levels and CAD severity, healthcare providers can enhance patient care and outcomes, especially in resource-limited settings. This present study yielded significant insights into the relationship between serum vitamin D levels and the severity of CAD. Moreover, we determined the prevalence of various established risk factors for CAD and explored the variations in their distribution between two distinct patient groups: those with non-severe CAD and those with severe CAD.

Among the 285 patients in our study, n=188 (65.95%) had non-severe CAD, whereas n=97 (34.05%) had severe CAD. A comparable distribution of CAD severity was presented in another Pakistani study [5]. In contrast, an Indian study found a higher proportion of severe CAD in the Indian population [21]. The discrepancy in CAD severity between studies may be attributed to differences in the prevalence of traditional cardiovascular risk factors among the study population.

Assessment of the demographic features of the study population indicated that participants with severe CAD had a higher mean age (59.24 years, SD ± 17.68) than patients with non-severe CAD (57.50 years, SD ± 14.60). Furthermore, CAD was more prevalent among male patients, accounting for 170 (59.65%) cases. These findings are consistent with previous research that reported comparable demographic trends in patients with CAD [9].

Notably, hypertension was the most common established risk factor in the study population, followed by diabetes mellitus, dyslipidemia, smoking history, and family history of CAD. Although patients with a family history of CAD, hypertension, diabetes mellitus, dyslipidemia, and smoking habits had a higher incidence of severe CAD, the difference did not reach statistical significance in comparison to those without these risk factors (Table 1). An identical distribution of predisposing factors is in line with previous studies [5,8,9,13].

Pearson's correlation analysis of variables of the present study demonstrates that lower vitamin D levels are strongly associated with more severe CAD, as reflected by higher Gensini scores (Table 2). Moreover, simple linear regression also confirmed this inverse correlation between the serum vitamin D level and CAD severity through the negative regression coefficient, which suggests that lower vitamin D levels are closely linked to higher Gensini scores, which in turn indicate more severe CAD (Table 3). Likewise, this correlation is supported by previous global research highlighting the potential role of vitamin D deficiency in the development and progression of CAD. A study from India demonstrated that serum vitamin D has a significant role in the pathogenesis and progression of CAD [7]. A study from Poland also reported the association between vitamin D deficiency and the development of CAD [9]. Another study from Egypt has recorded similar results, indicating that lower serum vitamin D levels are linked with increased CAD severity [10]. A systematic review of ten randomized controlled trials and three observational studies also found that vitamin D supplementation in cardiac patients could improve their outcomes, showing that lower vitamin D levels are associated with greater CAD severity [12]. Similarly, a study from Greece also backed the significant role of vitamin D in the progression of cardiovascular disease severity [14]. Moreover, an Iranian study has also validated the inverse relationship between vitamin D levels and the severity of CAD [16]. Likewise, a study from the United States has endorsed the role of serum vitamin D as an independent risk factor for CAD development and progression [17]. Our results align with previous research, confirming that lower vitamin D levels are a novel biomarker and risk factor for CAD severity in patients with MI.

The pathophysiological mechanisms underlying this association between vitamin D deficiency and CAD development and progression are multifaceted [1]. Vitamin D deficiency has been shown to promote atherosclerosis directly by enhancing cholesterol uptake by macrophages, foam cell formation, and subsequent plaque buildup in the coronary arteries [10]. Vitamin D deficiency has also been associated with lower levels of HDL and apolipoprotein A-1, which can further contribute to the development of atherosclerosis [12]. Additionally, vitamin D deficiency may contribute to increased blood pressure through influence on the renin-angiotensin-aldosterone system and atrial stiffness [13]. Lower vitamin D levels also lead to raised blood glucose levels due to increased insulin resistance and pancreatic beta-cell dysfunction [9,14]. Decreased levels of vitamin D also cause hyperparathyroidism, which mediates a detrimental impact on cardiac tissues. These mechanisms, along with the primary impact of vitamin D deficiency on atherosclerosis, further worsen the CAD severity [16-18].

The clinical implications of this study have great importance. This study's findings suggest that vitamin D levels may serve as a potential biomarker for CAD severity, which could guide risk assessment, treatment, and preventive strategies. Vitamin D supplementation may also improve cardiac health by mitigating the adverse effects of CAD. By integrating vitamin D level assessment and supplementation into clinical practice, clinicians may be able to reduce the burden of CAD and improve patient outcomes. Overall, this study highlights the importance of considering vitamin D levels in the management of patients with CAD and underscores the need for increased prevention strategies for CAD, particularly in developing countries. However, further research is needed to determine the causal relationships between vitamin D levels and CAD severity, as patients with more severe CAD could also lead to lower vitamin D through the associated lifestyle limitations.

Although this study's findings contribute to the growing body of evidence supporting the use of vitamin D as a predictor for CAD severity assessment. This study has several limitations, including its cross-sectional design, single-center setting, and limited sample size. Additionally, no adjustment of potential confounding factors via multivariate regression analysis and single-time-point vitamin D measurement may have influenced the results. Future research should focus on longitudinal studies to establish causality, interventional studies to determine the effect of vitamin D supplementation, and multi-center studies to enhance generalizability. Further investigation into the underlying mechanisms and integration of vitamin D levels into existing CAD risk scores may also improve predictive accuracy and clinical utility.

## Conclusions

This study presented a significant inverse correlation between serum vitamin D levels and CAD severity in patients with MI. Lower vitamin D levels were closely associated with more severe CAD, as indicated by higher Gensini scores. The results suggest that vitamin D deficiency may be a potential factor in CAD development and progression, underscoring its possible use as a risk indicator and diagnostic tool for high-risk patients. Considering the ease and cost-effectiveness of vitamin D level measurement, its application in cardiac risk evaluation is advisable, especially in resource-constrained settings. Timely interventions, including vitamin D supplementation and other cardiovascular risk-reducing strategies in cardiac patients with vitamin D deficiency, may enhance outcomes and lower mortality rates by slowing disease progression. Additional studies are needed to confirm the role of vitamin D in assessing CAD severity and to determine its role in guiding preventive approaches and reducing disease progression. Overall, this study further supports the role of vitamin D as a biomarker and risk factor in CAD severity assessment.

## Appendices

Appendix 1 

**Table 4:** Self-designed research proforma.

Self-Designed Research Proforma			
Parts	Research Questions	Options: write/tick the option	
Part A. (History, Physical Examination, and Medical Records)			
1. What is the age of the patient? (Years)			
2. What is the gender of the patient?		Male	Female
3. What are the presenting complaints of the patient?			
4. What is the duration of presenting complaints? (Minutes)			
5. Does the patient have a family history of Coronary Artery Disease?		Yes	No
6. Does the patient have a history of Diabetes Mellitus?		Yes	No
7. Does the patient have a history of Hypertension?		Yes	No
8. Does the patient have a history of Dyslipidemia?		Yes	No
9. Does the patient have a history of Smoking?		Yes	No
10. Does the patient have a history of Cardiac or any other Surgery?		Yes	No
11. Does the patient have a history of Past Treatment?		Yes	No
12. Does the patient have a history of any Chronic Disease other than those mentioned above?		Yes	No
13. What are the physical examination findings of the patient? (Vitals, General, and Systemic)			
14. What are the findings of the Past Medical Record?			
Part B. (Investigations Reports)			
1. What are the Electrocardiogram Findings?			
2. What is the Cardiac Biomarker Level (Troponin I) (ng/ml)?			
3. What are the Coronary Angiography Findings?			
4. What is the Gensini Score?			
5. What is the Severity of Coronary Artery Disease as per the Gensini Score?	Non-severe Coronary Artery Disease Group (Up to 50)	Severe Coronary Artery Disease Group (Above 50)	
6. What is the serum Vitamin D level (ng/mL)?			
7. What is the Vitamin D level status?	Normal (Above 30 ng/mL)	Deficiency (Less than 20 ng/mL)	
8. What is the Serum Lipid level (mg/dL)?			
9. What is the Lipid level status?	Normal (Less than 200 mg/dL)	Abnormal (Above 200 mg/dL)	
10. Is there any Abnormality in any of the following Investigations: C-Reactive Protein / Erythrocyte Sedimentation Rate / White Blood Cell Count/ Liver Function Tests / Renal Function Test?	Yes	No	
